# Updated mtCOI reference dataset for the *Bemisia tabaci* species complex

**DOI:** 10.12688/f1000research.12858.1

**Published:** 2017-10-13

**Authors:** Laura M. Boykin, Anders Savill, Paul De Barro

**Affiliations:** 1 School of Molecular Sciences and Australian Research Council Centre of Excellence in Plant Energy Biology, University of Western Australia, Crawley, Perth, WA, Australia; 2 CSIRO, Ecosciences Precinct, Brisbane, QLD, Australia

**Keywords:** species identification, whitefly, insect vector, mitochondrial cytochrome oxidase, DNA barcoding

## Abstract

Members of the whitefly *Bemisia tabaci *species complex cause millions of dollars of damage globally and are considered one of the world’s most invasive species. They are capable of causing extensive damage to major vegetable, grain legume and fiber crops. All member of the species complex are morphologically identical therefore, data from the partial mitochondrial cytochrome oxidase subunit I (mtCOI) gene sequence has been used to identify the various species. The current reference dataset that is widely used is found on the CSIRO data portal. However, the reference set stored on the CSIRO data does not include newly added sequences (2013-2017), therefore an updated reference dataset is needed.  All mtCOI data for the *Bemisia tabaci* species complex were downloaded on 22 May 2017 from GenBank and after quality checking, a dataset of 1,071 unique sequences and 696 base pairs was generated (https://doi.org/10.6084/m9.figshare.5437420.v1).

## Introduction

Members of the *Bemisia tabaci* (whiteflies) species complex are among the world’s most devastating insect pests and cause billions of dollars (US) of damage each year, leaving farmers in the developing world food insecure ( De Barro *et al.*, 2011). As a species complex with at least 34 members, identification is based on the use of the 657 bp portion of the 3’ end of the mitochondrial COI (mtCOI) ( Boykin *et al*., 2012, Boykin *et al*., 2013). In order to identify members of the complex correctly, a curated reference dataset is a useful resource. In 2012, a reference mtCOI dataset was made available on the CSIRO data portal ( De Barro & Boykin, 2012). Errors in the dataset were subsequently identified and so the dataset was updated on 15 May 2017 ( http://doi.org/10.4225/08/591a4018dfca8) ( De Barro & Boykin, 2017), but did not include new additions from GenBank (post 2012). Therefore, the dataset described herein represents the most up-to-date reference resource for members of the complex.

## Methods

The CSIRO dataset ( http://doi.org/10.4225/08/591a4018dfca8), updated 15 May 2017 was used as the starting point. The existing records were updated to include host plant data. New records post-2012 were then downloaded on 22 May 2017 directly from GenBank. All downloaded data was treated as follows:

1) Data was classified with BLAST using the new CSIRO reference data set

2) Sequences that caused gaps in the alignment were removed

2) Sequences that had stop codons present were removed

3) Clustal Omega ( Sievers & Higgins, 2014) was used for preliminary alignment and fine tuning of the alignment was carried out with MAFFT ( Katoh & Standley, 2013).

4) Duplicate sequences were then removed using BBMAP Dedupe ( Bushnell, 2017).

In addition, all MEAM2 sequences were removed as they have now been confirmed to be pseudogenes ( Tay *et al*., 2017).

## Data availability

The data referenced by this article are under copyright with the following copyright statement: Copyright: © 2017 Boykin LM et al.

Figshare: Dataset 1. mtCOI reference data for species ID of *Bemisia tabaci*. DOI: 10.6084/m9.figshare.5437420 ( Boykin *et al*., 2017)
