# Supplementary material for: Updated mtCOI reference dataset for the Bemisia tabaci species complex
Source: F1000Res. 2017 Oct 13;6:1835. [Version 1] doi: 10.12688/f1000research.12858.1 (PMC5680533; doi:10.12688/f1000research.12858.1)
Supplement: Peer review history

# Peer review: Referee response for version 1

**DOI:** 10.5256/f1000research.13935.r26987

**Published:** 2017-11-08

**Authors:** Sharad Saurabh, Manisha Mishra

**Affiliations:** 1 Molecular and Structural Biology Division, CSIR-Central Institute of Medicinal and Aromatic Plants, Lucknow, India; 2 Developmental Toxicology Division, CSIR-Indian Institute of Toxicology Research, Lucknow, India

Whitefly ( *Bemisia tabaci*) is becoming a global hazard for crop and ornamental plants. Identification of correct species is always better for the implication of best control strategy. In this regard, the effort made by Boykin et al for speedy and accurate identification of *B. tabaci* species complex is very significant. Additionally, the dataset developed with enriched quality is also very useful for whitefly biologist working in the area of evolution, mitochondrial genomics and crop management.

 All the bioinformatics tools used to generate this refined dataset are ideal to make such analysis.

We have read this submission. We believe that we have an appropriate level of expertise to confirm that it is of an acceptable scientific standard.

# Peer review: Referee response for version 1

**DOI:** 10.5256/f1000research.13935.r26988

**Published:** 2017-10-23

**Authors:** Renate Krause Sakate

**Affiliations:** 1 Department of Plant Protection, Faculty of Agronomic Sciences, São Paulo State University (UNESP), Botucatu, Brazil

The updated mtCOI reference dataset for the *Bemisia tabaci *species complex will add a valuable contribution to researches for a fast and accurate identification of members from the *B. tabaci *species complex based on the partial mitochondrial COI gene.

 The high quality data is easily accessible for download and gathers whiteflies collected globally. The availability of a reliable and updated reference dataset is an essential tool that will aid the scientific community to identify and classify correctly this  pest, the first step in the crop management against whiteflies.

I have read this submission. I believe that I have an appropriate level of expertise to confirm that it is of an acceptable scientific standard.
